# Synthesis of Uniform Alkane-Filled Capsules with a High Under-Cooling Performance and Their Real-Time Optical Properties

**DOI:** 10.3390/polym11020199

**Published:** 2019-01-24

**Authors:** Jinyun Liu, Yong Wu, Wen Zhang, Jiawei Long, Ping Zhou, Xi Chen

**Affiliations:** 1 Key Laboratory of Functional Molecular Solids, Ministry of Education, College of Chemistry and Materials Science, Anhui Normal University, Wuhu 241002, China; wy2425@ahnu.edu.cn (Y.W.); zw1130@ahnu.edu.cn (W.Z.); jwlong@ahnu.edu.cn (J.W.L.); 2 Institute of Intelligent Machines, Chinese Academy of Sciences, Hefei 230031, China; lenazp@mail.ustc.edu.cn; 3 Department of Chemistry, University of Science and Technology of China, Hefei 230026, China; 4 Beckman Institute for Advanced Science and Technology, University of Illinois at Urbana-Champaign, Urbana, IL 61801, USA; xichen9112@gmail.com

**Keywords:** capsules, under-cooling, preparation, thermal properties

## Abstract

Encapsulating under-cooling materials has been a promising strategy to address the compatibility issue with a surrounding matrix. Herein, we present the synthesis of a uniform alkane-infilled capsule system that shows obvious under-cooling properties. As demonstrating examples, *n*-hexadecane was selected as a liquid alkane and *n*-eicosane as a solid in our systems as core materials via in-situ polymerization, respectively. The under-cooling properties of capsules were investigated using differential scanning calorimetry, real-time optical observations with two polarizers, and molecular modeling. The *n*-hexadecane encapsulated capsules exhibited a large under-cooling temperature range of 20 °C between melt and crystallization, indicating potential applications for structure-transformation energy storage. In addition, molecular modeling calculations confirmed that the solid forms of *n*-hexadecane and *n*-eicosane are more stable than their liquid forms. From liquid to solid form, the *n*-hexadecane and *n*-eicosane release energies were 4.63 × 10^3^ and 4.95 × 10^3^ J·g^−1^, respectively.

## 1. Introduction

Stimuli (e.g., heat, mechanical force, and photo radiation) responsive phase-change materials (PCMs) have been of great interest because of their versatile properties including optics and mechanics [1,2,3,4,5]. Among them, under-cooled materials that remain liquid at the temperature below their freezing point without turning into solid phase, and crystallize by external stimuli, will release latent heat at lower temperatures [6,7]. These properties enable, in the under-cooled materials, a potential for many applications such as energy absorbance and energy relieving applications such as smart hand warmers, thermally enhanced fabrics and textile fibers, light weighted energy saving and temperature controllable buildings, and heat insulation foams [8,9,10]. It has been reported that long chain *n*-alkanes, as an important organic PCMs, have potential for such applications due to their chemical stability, non-corrosive properties, and high energy storage capacity [11,12]. However, there are still many issues that severely limit the direct use of alkanes, such as the poor compatibility with other materials, limited heat transfer area, and interference from surroundings.

Encapsulating long chain *n*-alkanes into capsules is a potentially effective approach to address those issues [13,14,15,16]. This leads to three points: firstly, the compatibility between capsules and the matrix can be tuned by adjusting the shell composition and surface chemistry; secondly, the distribution of alkanes as functional materials can be well controlled; finally, the mechanical strength is enhanced. Recently, alkane encapsulation has attracted attention, especially for the exploration of optimal core shell integration [17,18,19,20,21]. Yang et al. successfully encapsulated octadecane with a melamine-formaldehyde resin shell via an oil-in-water emulsion technique [22]. Sari et al. prepared polymethylmetracrylate microcapsules containing *n*-octacosane for energy storage [23]. Yang et al. reported a phase separation method to fabricate *n*-tetradecane microcapsules with three different shells: acrylonitrile-styrene copolymer, acrylonitrile-styrene-butadiene copolymer, and polycarbonate [24]. Additionally, poly(urea-formaldehyde) has been applied as a shell material for encapsulating epoxy resin and dicyclopentadiene for self-healing [25,26].

Until now, exploring an optimal core shell integration that possesses good compatibility, high structure strength, and a large and suitable phase-change temperature range for specific applications (e.g., around room temperature) remains a great challenge. Moreover, the controllable encapsulation and the heat-dependent phase change behaviors are still unclear.

Here, we present an alkane-encapsulated poly(urea-formaldehyde) microcapsule system, which was prepared via an in-situ polymerization method. For demonstrations, n-hexadecane and n-eicosane were employed as typical liquid (at room temperature) and solid long chain alkanes as core materials of microcapsules, respectively. In our investigation, (i) the material system of the capsules such as the shell are different from previous reports; (ii) the melting-crystallizing behaviors demonstrated through real-time optical polarizer observations are reported here for the first time; (iii) we provide a generalized microcapsule system which is applicable for many possible core materials. The thermal stability and the optimal process for high-quality encapsulation have been investigated. Moreover, their temperature-dependent phase-change properties and potential mechanisms are demonstrated. The presented high-performance capsule systems with good super-cooling performance and stable reversibility are expected to be applicable in many fields, including energy storage and conversion devices, thermally controllable smart mechanical components, and low-energy consumption houses.

## 2. Experimental

### 2.1. Microcapsule Preparation

First, 1.25 g of urea, 0.25 g of resorcinol, and 0.125 g of NH_4_Cl were added to a solution of 50 mL of de-ionized water and 28 mL of 2.5 wt % ethylene-maleic anhydride (EMA) copolymer under stirring. Second, the pH value of the above solution was adjusted to 3.5 by triethanolamine (TEA), and the temperature was then elevated to 55 °C. Once the temperature was stabilized, the solution was mechanically stirred at a particular rotation speed directly following the addition of 4 g of *n*-hexadecane (or pre-melted *n*-eicosane). After the core materials were added to the solution, an oil-in-water emulsion was formed by mechanical stirring. The oil separated into small droplets, and was stabilized by surfactant in this system. Next, after stirring for 30 min, the polymerization was activated when 3.2 g of 37 wt % formaldehyde solution was added drop by drop. At this point, the poly(urea-formaldehyde) formed at the oil and water interface to encapsulate the oil. After stirring for another 2 h, the solution was cooled down to room temperature. The microcapsules formed, which were floating in the solution due to their low density, were separated from the aqueous solution and washed with de-ionized water ultrasonically 3 times. At last, the samples were dried in a freeze-drying system.

### 2.2. Characterization

The surface morphology of microcapsules was observed using a Hitachi S-4700 scanning electron microscope (SEM, Tokyo, Japan). Prior to characterization, the specimen was coated by sputtering a ~2 nm thick platinum layer for electrical conductivity improvement. The core shell structure of the microcapsule was characterized by a JEOL 2010 LaB6 transmission electron microscopy (TEM, Tokyo, Japan) operating at 200 kV, and a Leica DMR optical microscopy. Differential scanning calorimetry (DSC) and thermogravimetric analysis (TGA) were performed by a Mettler-Toledo DSC821e (Zurich, Switzerland) and a TGA/SDTA 851e (Zurich, Switzerland), respectively. TGA was conducted at a heating rate of 10 °C·min^−1^ under flowing N_2_. To investigate the real-time phase-change behaviors, a Bruker Hyperion microscope equipped with two additional polarizers was employed for continuous observation. Each polarizer was fixed above or below the samples. A glass slide loaded with capsules was placed on a heating holder (between two polarizers) where the temperature was controlled by a N_2_ liquid flow system. The heating–cooling rate was 5 °C·min^−1^ under the temperatures from −15 to 45 °C for *n*-hexadecane and from 20 to 65 °C for *n*-eicosane, respectively.

## 3. Results and Discussion

**Figure 1:**
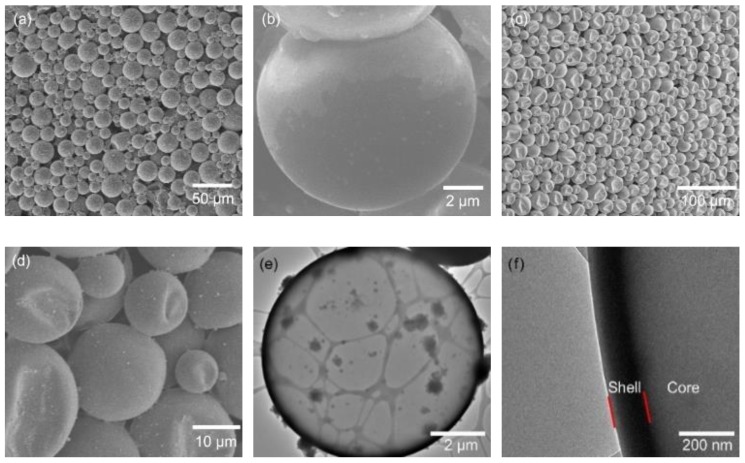

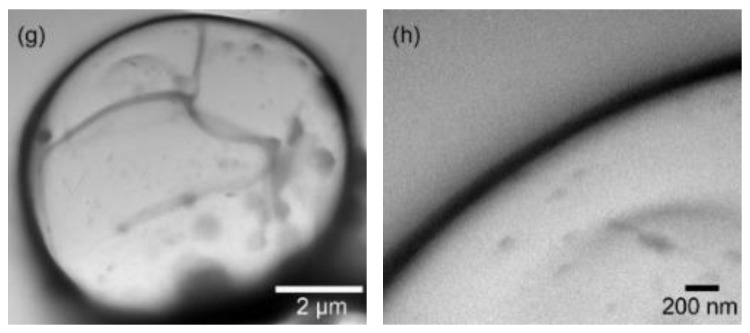
SEM images of the (**a**,**b**) *n*-hexadecane- and (**c**,**d**) *n*-eicosane-encapsulated microcapsules. TEM images of (**e**,**f**) *n*-hexadecane- and (**g**,**h**) *n*-eicosane-encapsulated capsules. The core-shell structure is marked in (**f**).

The SEM images of *n*-hexadecane- and *n*-eicosane-encapsulated microcapsules are shown in Figure 1. The capsules are in a size distribution of about 10–35 µm. In Figure 1a,b, the *n*-hexadecane-encapsulated capsules show a spherical structure since there is no phase change (melting point of *n*-hexadecane is 18 °C). In contrast, the *n*-eicosane-encapsulated capsules (Figure 1c,d) show irregular shapes rather than perfect spheres. It is ascribed to the solid phase nature of *n*-eicosane at room temperature (melting point = 36–38 °C), because it was melted into liquid prior to encapsulation. A remarkable volume change occurred when the core cooled down and converted from liquid to solid. A high-quality shell, which is demonstrated by TEM images (Figure 1e–h), was essential for preventing capsules from breaking and core-leaking. The shell thicknesses of both *n*-hexadecane- and *n*-eicosane-encapsulated capsules were 100–160 nm. No cracks and pores were observed, indicating a strong shell for core protection. The size distribution of capsules was observed using an optical microscope, as shown in Figure S1. The liquid core in *n*-hexadecane-encapsulated capsules shows a smooth and transparent profile. whereas the solid core in *n*-eicosane-encapsulated capsules is much rougher and contains shadows.

**Figure 2:**
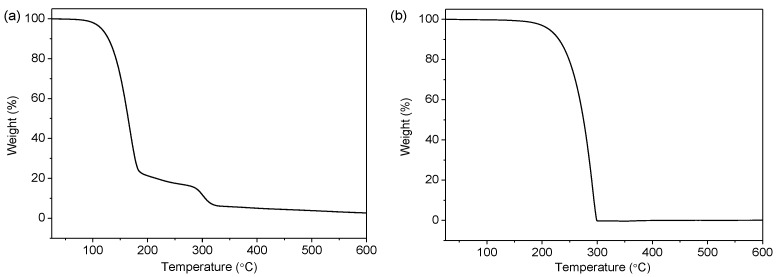
TGA curves of (**a**) *n*-hexadecane- and (**b**) *n*-eicosane-encapsulated capsules at a heating rate of 10 °C min^-1^ in nitrogen flow.

The core contents of the *n*-hexadecane and *n*-eicosane-encapsulated microcapsules were characterized using TGA, as shown in Figure 2. A rapid weight drop was observed, which indicates that the capsules break at a narrow temperature range, exhibiting a uniform shell strength. On the other hand, since a wide size distribution of capsules would lead to a mild weight drop due to the shell strength difference, it is reasonable to assume that the prepared capsules are in a narrow size distribution, which is in good agreement with the SEM and optical microscope observations shown above. Figure 2a shows the TGA curve of the *n*-hexadecane-encapsulated capsules, which displays two periods of weight losses during the heating process. The first loss from about 130 °C can be ascribed to the evaporation of the *n*-hexadecane, while the second loss at about 300 °C is due to the decomposition of the poly(urea-formaldehyde) shell. As for the *n*-eicosane-encapsulated capsules, there is one obvious weight loss in the TGA curve, as shown in Figure 2b. This is attributed to the relatively high evaporation temperature of *n*-eicosane (boiling point = ~343 °C), which is close to the decomposition of capsule shell. It is noted that, in the TGA curves, the residual weights are as low as 2% at 600 °C. The tiny remained weights perhaps are from the materials carbonized at high temperature under the nitrogen gas during measurements.

**Figure 3:**
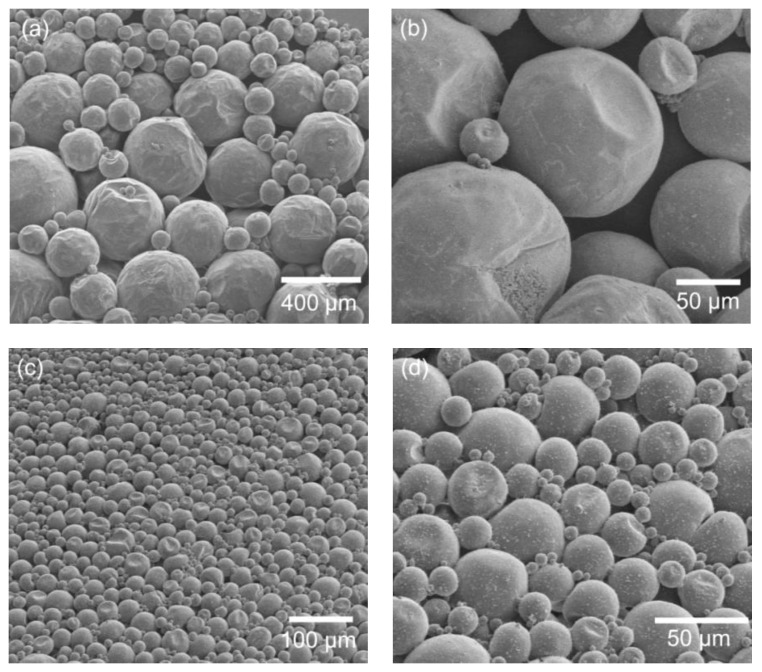
SEM images of *n*-eicosane-encapsulated microcapsules prepared at different emulsification speeds: (**a**,**b**) 1200 rpm and (**c**,**d**) 2000 rpm. The polymerization time and surfactant volume were 2 h and 28 mL, respectively.

Since the preparation conditions of microcapsules are essential for both high-quality control and future investigation extensions, a series of factors were investigated, including the emulsification speed, polymerization time, and surfactant amount. First, taking *n*-eicosane-encapsulated capsules as an example for demonstration, the SEM images of the capsules prepared at different emulsification speeds ranging from 1200 to 2000 rpm are shown in Figure 3. The diameter change among different samples is obvious, indicating an emulsification speed-dependent size distribution effect. This shows that the capsule size is inversely related to the emulsification speed. At an emulsification speed of 1200 rpm, the diameter of *n*-eicosane-encapsulated capsules is ca. 150 µm (Figure 3a,b), while at 2000 rpm the diameter reduces to about 50 µm (Figure 3c,d). Increasing to a 3000 rpm emulsification speed, the size continuously decreases to about 10–30 µm. Meanwhile, the size distribution becomes narrow depending on the speed increases, showing a more uniform distribution at higher speeds. The mechanism by which emulsification speed influences the capsule size can be explained as follows. The droplet size of the oil phase in the solution is affected by the speed. The higher speed is, the smaller drops will be. When the shell is polymerized, which is composed of poly(urea-formaldehyde) at the oil and water interface, the capsule size is determined in terms of the size of the oil-phase droplets.

**Figure 4:**
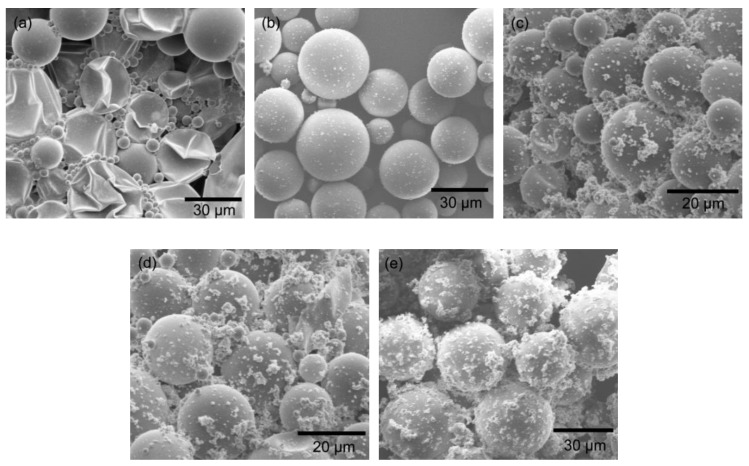
SEM images of microcapsules (*n*-hexadecane as core materials) prepared under different polymerization times: (**a**) 1 h, (**b**) 2 h, (**c**) 3 h, (**d**) 4 h, and (**e**) 5 h. The emulsification speed and surfactant volume were kept at 3000 rpm and 28 mL, respectively.

Figure 4 shows the SEM image of the *n*-hexadecane-encapsulated microcapsules obtained under different polymerization times (1–5 h). It is found that the polymerization time exhibits a great influence to the shell morphology. If the polymerization time is relatively short (1 h), a large portion of capsules are broken (Figure 4a), which may be ascribed to the thin shell thickness and weak structure with poor mechanical strength because of the uncompleted polymerization of the poly(urea-formaldehyde) shell. Meanwhile, the yield of capsules in this condition is as low as 60%. In contrast, extending the polymerization period allows the capsules more time to maintain a narrower size distribution. This phenomenon is similar to an Ostwald ripening mechanism during crystal growth. The Ostwald ripening considers that larger particles are more energetically favored than smaller particles, and the small crystals or sol particles would dissolve and redeposit onto larger crystals or sol particles, resulting in a thermodynamically driven spontaneous process. In addition to this, it is reasonable to assume that the shell becomes thick because of the increased time allowed for polymerization, leading to the enhancement of the shell strength. When employing two-hour polymerization, the yield of capsules with a high-quality shell reaches above 95%. However, as the polymerization time continuously increases, the surface of the microcapsules becomes rough with many particles adhered to them (Figure 4c–e). When the reaction time is too long, some of the capsules break because of the mechanical stirring. The broken fragments then adsorb on the surface of capsules, resulting in a rough surface of the capsules obtained after a long period of polymerization.

**Figure 5:**
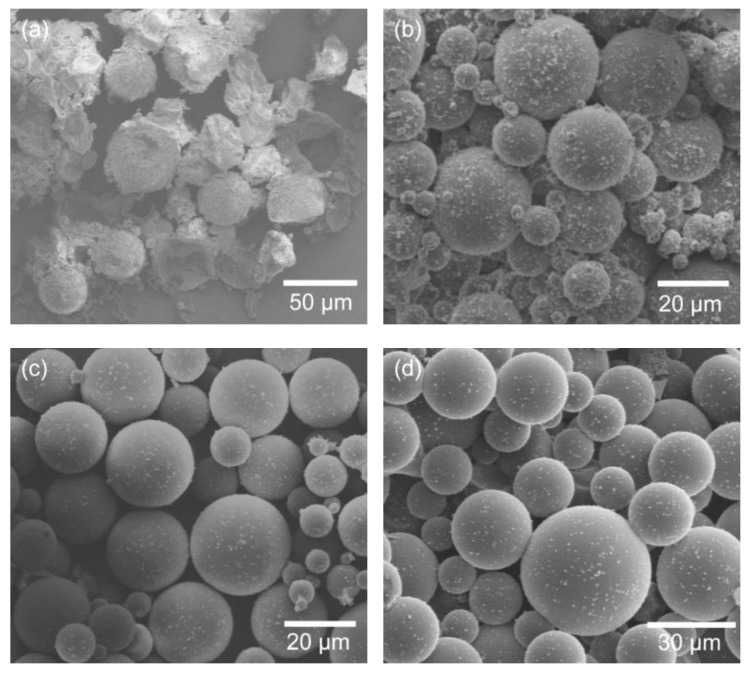
SEM images of *n*-hexadecane-encapsulated capsules obtained under an emulsification speed of 3000 rpm and a polymerization time of 2 h using different volumes of EMA as surfactant: (**a**) 15, (**b**) 20, (**c**) 28, and (**d**) 35 mL.

The relationship between the amount of surfactant (EMA) and the capsule quality was demonstrated, as shown in Figure 5. It is known that the surfactant (EMA in our study) is crucial for emulsification, as it enables the organic core material droplets to remain stable in aqueous solution without agglomeration. In Figure 5, the size distribution becomes narrow as the surfactant increases. When the EMA was continuously increased (Figure 5d), there was no obvious difference in the surface morphology of the microcapsule. However, excessive surfactant made the entire solution very stable and converted into only one phase, resulting in a great difficulty for microcapsule collection, which may reduce the final yield of microcapsules. These investigations lead to the conclusion that a suitable amount of surfactant is necessary for both high-quality encapsulation and large yield.

**Figure 6:**
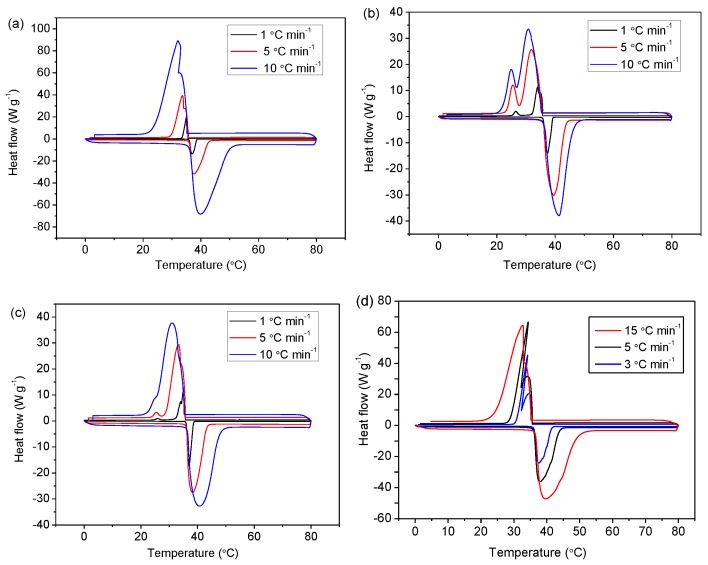
DSC curves of *n*-eicosane-encapsulated capsules with different sizes: (**a**) ~150 µm, (**b**) ~50 µm, and (**c**) ~20 µm. The heating–cooling rates vary at 1, 5, and 10 °C·min^−1^. (**d**) DSC curves of bulk *n*-eicosane chemical without encapsulation, with heating–cooling rates of 3, 5, and 15 °C·min^−1^.

DSC measurements were conducted for evaluating the phase-change behaviors of the microcapsules, as shown in Figure 6. We find that both the endothermic and exothermic peaks become sharp as the heating–cooling rate decreases; moreover, a slower heating–cooling rate leads to a narrower under-cooling temperature range (the temperature between the endothermic and exothermic peaks). Another observation was that, as the capsule size reduced to about 50 and 20 µm (DSC curves of 20 µm *n*-hexadecane-encapsulated capsules are shown in Figure S2), an additional exothermic peak appeared (Figure 6b,c), which was perhaps attributed to homogeneous nucleation [27]. This may indicate enhanced super-cooling compared to the large capsules (~150 µm) shown in Figure 6a and the bulk *n*-eicosane without encapsulation (Figure 6d). In the large capsules, more particle seeds would exist, leading to an easier nucleation, and thereby the super-cooling performance would be reduced. In addition, as can be seen in Figure 6a–c, the temperature ranges of DSC measurements are the same, and the curve profiles are similar, indicating a good reversibility of the phase-change. In other words, the capsules remain stable over the temperature window of 0–80 °C.

From the DSC results shown above, we propose two potential mechanisms responsible for the phase-change behaviors of microcapsules under different thermal conditions. First, during the cooling process, the different nucleation kinetics of core materials might relate to the capsule size, since the smaller capsules (50 and 20 µm) undergo partially homogeneous nucleation rather than total heterogeneous nucleation of larger capsules (150 µm). Second, the different degrees of impurities inside microcapsules could be a reason for whether homogeneous or heterogeneous nucleation dominates. The impurities inside the capsules provide nucleation seeds to stimulate heterogeneous nucleation, which initiates at a higher temperature compared to homogeneous nucleation. In contrast, capsules encapsulated with a highly pure core material will lead to a homogeneous nucleation-dominated crystallization, showing a large under-cooling temperature range since the melting point of the core material remains at the same temperature.

The molecular models of *n*-hexadecane and *n*-eicosane are displayed in Figure S3, where the hydrogen atoms are in red and the carbon atoms are in grey. Tables S1 and S2 show the calculated free energies for hexadecane and eicosane with different calculation steps, where the liquid molecules are embedded in the sizes of 50 Å × 50 Å × 50 Å, 55 Å × 55 Å × 55 Å, and 60 Å × 60 Å × 60 Å, respectively. With the increasing of the calculation step, the energies for *n*-hexadecane and *n*-eicosane exhibit stabilized behaviors. The energies of solid forms for *n*-hexadecane and *n*-eicosane are −7.79 × 10^4^ and −7.77 × 10^4^ J·g^−1^ at 5000 steps, respectively. The energies of liquid forms for *n*-hexadecane and *n*-eicosane are −3.16 × 10^4^ and −2.67 × 10^4^ J·g^−1^ at 5000 steps within 50 Å × 50 Å × 50 Å, respectively. This indicates that the solid forms for *n*-hexadecane and *n*-eicosane are more stable than their liquid forms. From liquid to solid form, the structures of *n*-hexadecane and *n*-eicosane release energies of 4.63 × 10^3^ and 4.95 × 10^3^ J·g^−1^, respectively.

**Figure 7:**
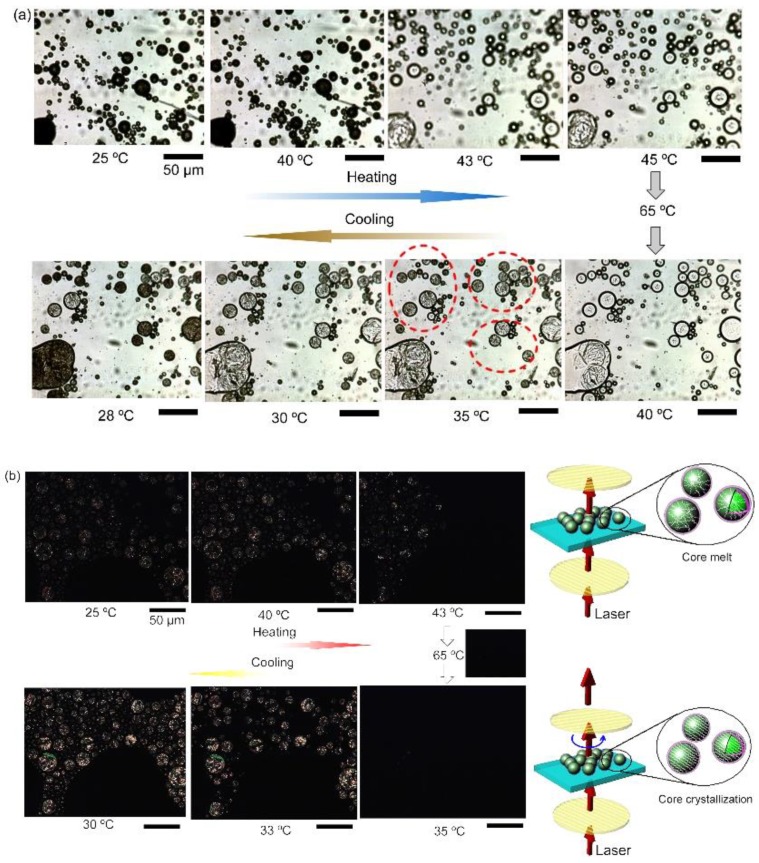
Real-time optical images of the *n*-eicosane-encapsulated microcapsules during heating–cooling process under transmission mode (**a**) without and (**b**) with polarizers. The heating–cooling rate is 5 °C·min^−1^. The circuit (in red) marked in (**a**) shows the crystals appearing inside capsules. The insets in (**b**) illustrate the melting and crystallizing differences of the capsules and the laser routes, respectively. The inserted scale bars stand for 50 μm.

**Figure 8:**
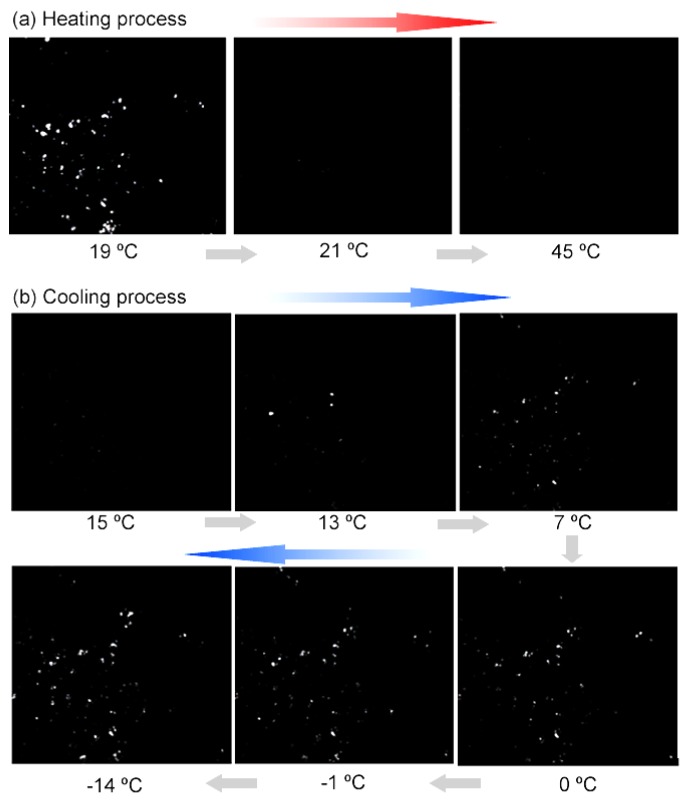
Real-time optical images of the *n*-hexadecane-encapsulated microcapsules during the heating–cooling under transmission mode of microscope equipped with polarizers.

For experimental investigations to observe real-time phase-change behavior, the microcapsules were placed under a continuous heating–cooling process and were monitored with an optical microscope equipped with two polarizers. In Figure 7a, when no polarizer was used, we can see the *n*-eicosane-encapsulated capsules changed from dark to transparent when heating at about 40 °C, indicating a melting process of core materials. As the temperature decreases, solids inside the capsules appear at around 35 °C, representing a crystallization process. As shown in Figure 7b, after two polarizers were introduced into the optical system, we can see that, in the heating process, the capsules become dark at 40 °C. It should be mentioned that, prior to the heating process, the two polarizers were adjusted to make the laser unable to pass through. Thus, once the core materials transform from an order-arranged solid structure to a random liquid state, meaning there is no grating-like crystal plane arrangement for laser deflection, no laser can be detected above the upper polarizer. Interestingly, during the cooling process, when the temperature decreases to 33–35 °C, some bright spots show up, indicating a phase-change process of crystallization, which is in good agreement with the exothermal peak location in DSC curves. Real-time phase-change observations were also conducted for *n*-hexadecane-encapsulated capsules, as shown in Figure 8. Two main periods of bright spots appearing during the cooling process are found to be at about 10 and 0 °C, which are perhaps due to the different structure transformation stages. Conversely, the darkening of the capsules at ~20 °C, confirming an under-cooling temperature.

Furthermore, during the structure transformation, in most cases, the large and small capsules change at the same time. There is no remarkable relationship between the capsules’ size and their structure transformation temperature during real-time observations. Because of that, the different degrees of purity inside capsules could play a more important role in contributing to the under-cooling performance compared to the capsule size. This is crucial for under-cooling applications because the bulk materials prior to encapsulation are easily impacted by impurities from surroundings; however, once they are encapsulated into an appropriate microcapsule system within a relatively isolated environment, the under-cooling performance can be substantially enhanced. It should be noted that, even though the capsule size does not have a critical influence on the under-cooling properties for the alkenes-poly(urea-formaldehyde) capsule system, the smaller size is still preferred for potential engineering applications. From our investigations, the larger capsules show relatively poor under-cooling performance, which is ascribed to the higher possibility of impurities being present in the core materials during the fabrication process.

**Figure 9:**
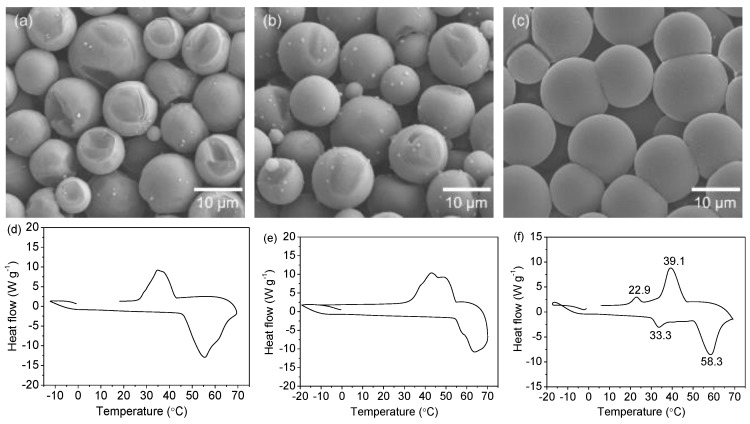
SEM images of the microcapsules encapsulated with (**a**) *n*-docosane, (**b**) *n*-hexacosane, and (**c**) a mixture of *n*-docosane and *n*-hexacosane (weight ratio = 1:1). Corresponding DSC curves (at a scanning rate of 10 °C·min^−1^) of the capsules encapsulated with (**d**) *n*-docosane, (**e**) *n*-hexacosane, and (**f**) a mixture of *n*-docosane and *n*-hexacosane.

With an encapsulation process similar to that of *n*-eicosane, a series of *n*-alkane-filled microcapsules were fabricated, as shown in Figure 9, showing a good generalization of the presented microcapsule system. As seen in the SEM images (Figure 9a–c), the microcapsules exhibit a smooth morphology with no cracks, along with a narrow size distribution. In DSC curves (Figure 9d–f), we can see obvious under-cooling performance. The extent of their thermal behaviors will be investigated further. A very interesting point is that in the case of microcapsules encapsulated with the mixture of *n*-docosane and *n*-hexacosane, multiple phase changes originating from endothermal and exothermal processes can be observed, which provides a potential opportunity for studying the thermodynamics of mixed systems under a confined micro-environment.

## 4. Conclusions

In summary, we have demonstrated a long chain alkane-encapsulated microcapsule system that possesses obvious under-cooling properties. *n*-Hexadecane as a representative liquid long-chain *n*-alkane and *n*-eicosane as a solid at room temperature were employed as core materials for encapsulation via an in-situ polymerization. The phase-change behaviors of capsules were investigated through real-time optical observations and DSC, and *n*-hexadecane-encapsulated microcapsules exhibited a high under-cooling performance of about 20 °C. Molecular modeling demonstrates that from liquid to solid form, the structures of *n*-hexadecane and *n*-eicosane release energies of 4.63 × 10^3^ and 4.95 × 10^3^ J·g^−1^, respectively. We believe these findings will be of great significance for novel phase-change capsule system design. Additionally, the presented microcapsules associated with the in-situ polymerization method will find applications in energy storage and release fields, mechanics, and optics modulation systems.

## Supplementary Materials

The supplementary materials are available online at http://www.mdpi.com/2073-4360/11/2/199/s1.

## References

[1] Huang J., Heise A. (2013). Stimuli Responsive Synthetic Polypeptides Derived From N-Carboxyanhydride (NCA) Polymerisation. Chem. Soc. Rev..

[2] Heine T. (2015). Transition Metal Chalcogenides: Ultrathin Inorganic Materials with Tunable Electronic Properties. Acc. Chem. Res..

[3] Dhaidan N.S., Khodadadi J.M. (2015). Melting and Convection of Phase Change Materials in Different Shape Containers: A Review. Renew. Sustain. Energy Rev..

[4] Qiu X.L., Lu L.X., Chen Z.Z. (2015). Preparation and Characterization of Flame Retardant Phase Change Materials by Microencapsulated Paraffin and Diethyl Ethylphosphonate with Poly(methacrylic acid-co-ethyl methacrylate) Shell. J. Appl. Polym. Sci..

[5] Choubineh N., Jannesari H., Kasaeian A. (2019). Experimental Study of the Effect of Using Phase Change Materials on the Performance of an Air-Cooled Photovoltaic System. Renew. Sustain. Energy Rev..

[6] Shao Z., Gopinadhan M., Kumar G., Mukherjee S., Liu Y.H., O’Hern C.S., Schroers J., Osuji C.O. (2013). Size-Dependent Viscosity in the Super-Cooled Liquid State of a Bulk Metallic Glass. Appl. Phys. Lett..

[7] Gucsik A., Tsukamoto K., Nishido H., Miura H., Kayama M., Ninagawa K., Kimura Y. (2012). Cathodoluminescence Microcharacterization of Forsterite in the Chondrule Experimentally Grown under Super Cooling. J. Lumin..

[8] Lu N., Sun D.F., Zhang C.K., Jiang N., Shang K.F., Bao X.D., Li J., Wu Y. (2018). CO_2_ conversion in non-thermal plasma and plasma/g-C3N4 catalyst hybrid processes. J. Phys. D Appl. Phys..

[9] Zhao C.Y., Zhang G.H. (2011). Review on Microencapsulated Phase Change Materials (MEPCMs): Fabrication, Characterization and Applications. Renew. Sustain. Energy Rev..

[10] Tyagia V.V., Kaushika S.C., Tyagib S.K., Akiyama T. (2011). Development of Phase Change Materials Based Microencapsulated Technology for Buildings: A Review. Renew. Sustain. Energy Rev..

[11] He F., Wang X.D., Wu D.Z. (2015). Phase-Change Characteristics and Thermal Performance of Form-Stable n-Alkanes/Silica Composite Phase Change Materials Fabricated by Sodium Silicate Precursor. Renew. Energy.

[12] Warzoha R.J., Fleischer A.S. (2014). Improved Heat Recovery From Paraffin-Based Phase Change Materials Due to the Presence of Percolating Graphene Networks. Int. J. Heat Mass Transf..

[13] Chai L.X., Wang X.D., Wu D.Z. (2015). Development of Bifunctional Microencapsulated Phase Change Materials with Crystalline Titanium Dioxide Shell for Latent-Heat Storage and Photocatalytic Effectiveness. Appl. Energy.

[14] Chaiyasat P., Suzuki T., Minami H., Okubo M. (2009). Thermal Properties of Hexadecane Encapsulated in Poly(divinylbenzene) Particles. J. Appl. Polym. Sci..

[15] He F., Wang X.D., Wu D.Z. (2014). New Approach for Sol-Gel Synthesis of Microencapsulated n-Octadecane Phase Change Material with Silica Wall Using Sodium Silicate Precursor. Energy.

[16] Nan G.H., Wang J.P., Wang Y., Wang H., Li W., Zhang X.X. (2014). Preparation and Properties of Nanoencapsulated Phase Change Materials Containing Polyaniline. Acta Phys.-Chim. Sin..

[17] Sari A., Alkan C., Bilgin C. (2014). Micro/Nano Encapsulation of Some Paraffin Eutectic Mixtures with Poly(methyl methacrylate) Shell: Preparation, Characterization and Latent Heat Thermal Energy Storage Properties. Appl. Energy.

[18] Rao Z.H., Huo Y.T., Liu X.J. (2014). Dissipative Particle Dynamics and Experimental Study of Alkane-Based Nanoencapsulated Phase Change Material for Thermal Energy Storage. RSC Adv..

[19] Daver H., Algarra A.G., Rebek J., Harvey J.N., Himo F. (2018). Mixed Explicit-Implicit Solvation Approach for Modeling of Alkane Complexation in Water-Soluble Self-Assembled Capsules. J. Am. Chem. Soc..

[20] Chen Z., Zhao Y.L., Zhao Y., Thomas H., Zhu X.M., Moller M. (2018). Inclusion of Phase-Change Materials in Submicron Silica Capsules Using a Surfactant-Free Emulsion Approach. Langmuir.

[21] Journey S.N., Teppang K.L., Garcia C.A., Brim S.A., Onofrei D., Addison J.B., Holland G.P., Purse B.W. (2018). Mechanically Induced Pyrogallol[4]arene Hexamer Assembly in the Solid State Extends the Scope of Molecular Encapsulation. Chem. Sci..

[22] Cao F.Y., Yang B. (2014). Supercooling Suppression of Microencapsulated Phase Change Materials by Optimizing Shell Composition and Structure. Appl. Energy.

[23] Sari A., Alkan C., Karaipekli A., Uzun O. (2009). Microencapsulated *n*-Octacosane as Phase Change Material for Thermal Energy Storage. Sol. Energy.

[24] Yang R., Zhang Y., Wang X., Zhang Y.P., Zhang Q.W. (2009). Preparation of n-Tetradecane-Containing Microcapsules with Different Shell Materials by Phase Separation Method. Sol. Energy Mater. Sol. Cells.

[25] Blaiszik B.J., Caruso M.M., McIlroy D.A., Moore J.S. (2009). Microcapsules Filled with Reactive Solutions for Self-Healing Materials. Polymer.

[26] Brown E.N., White S.R., Sottos N.R. (2004). Microcapsule Induced Toughening in a Self-Healing Polymer Composite. J. Mater. Sci..

[27] Tang X.F., Li W., Zhang X.X., Shi H.F. (2014). Fabrication and Performances of Microencapsulated *n*-Alkanes with Copolymers Having n-Octadecyl Side Chains as Shells. Ind. Eng. Chem. Res..

