# Supplementary material for: Synthesis of Uniform Alkane-Filled Capsules with a High Under-Cooling Performance and Their Real-Time Optical Properties
Source: Polymers (Basel). 2019 Jan 24;11(2):199. doi: 10.3390/polym11020199 (PMC6418806; doi:10.3390/polym11020199)
Supplement: Supplementary file 1 [file polymers-11-00199-s001.pdf]

Supporting Information

Synthesis of uniform alkane-filled capsules with a high under-cooling performance and their real-time optical properties

Jinyun Liu [1,*], Yong Wu [1], Wen Zhang [1], Jiawei Long [1], Ping Zhou [2,3], Xi Chen [4]

[1] Key Laboratory of Functional Molecular Solids, Ministry of Education, College of Chemistry and Materials Science, Anhui Normal University, Wuhu, Anhui 241002, P.R. China

[2] Institute of Intelligent Machines, Chinese Academy of Sciences, Hefei 230031, P.R. China

[3] Department of Chemistry, University of Science and Technology of China, Hefei, Anhui 230026, P.R. China

[4] Beckman Institute for Advanced Science and Technology, University of Illinois at Urbana-Champaign, Urbana, IL 61801, United States.

[*] Corresponding authors: J.Y.L. (jyliu@iim.ac.cn).

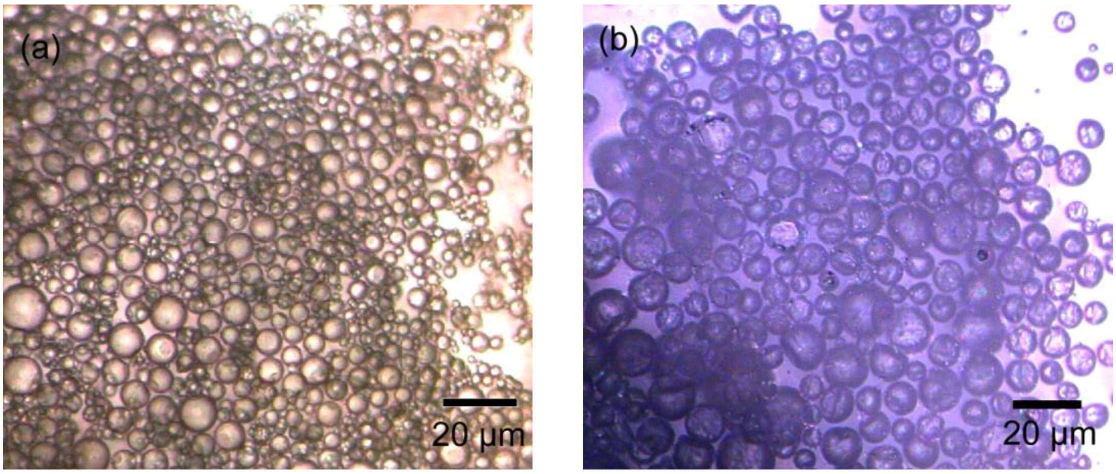

Figure S1. Optical images of (a) *n*-hexadecane- and (b) *n*-eicosane-encapsulated capsules.

Figure S2 displays the DSC curves of n-hexadecane-encapsulated microcapsules with a size of about 20 μm. They exhibit a similar heating-cooling rate-dependent trend of melting and crystallizing kinetics compared to the capsules encapsulated with n-eicosane. In contrast to the *n*-eicosane-encapsulated capsules, we can see there are three exothermal peaks in cooling process. The first exothermal peak at *ca.* 14.3 °C represents the first phase transition caused by a surface-induced heterogeneous nucleation, while the second one located at about *ca.* 8.7 °C indicates a metastable rotator phase structure formation [1]. The last exothermal peak at about 1.2 °C can be ascribed to the solid-to-solid homogenous structure transformation that occurs around this temperature [2,3]. Since the endothermic peak is located at 20 °C, the maximal temperature difference reaches up to 18 °C, exhibiting a good under-cooling performance.

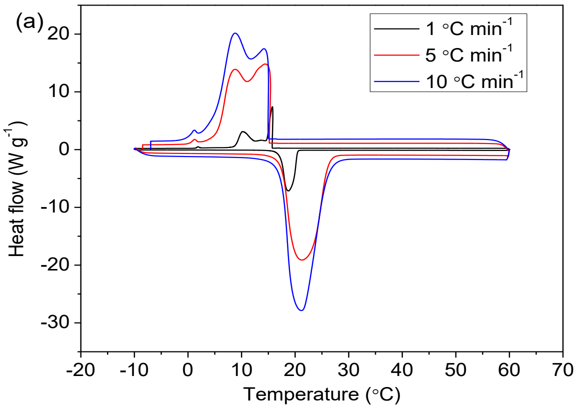

Figure S2. DSC curves of the n-hexadecane-encapsulated microcapsules (~20 μm diameter) under different heating-cooling rates.

Phase change energy modelings: We used the Sander module of Amber and the GAFF (General AMBER force field) to calculate the free energies and run the energy minimization of *n*-hexadecane and *n*-eicosane in solid and liquid forms, respectively. Amber is a widely used software package for running molecule simulations, which has been in development for many years. The main software package for molecule simulations in Amber is the Simulated Annealing with NMR-Derived Energy Restraints (SANDER), which is the central simulation program and provides facilities for energy minimizing and molecular dynamics with a wide variety of options.

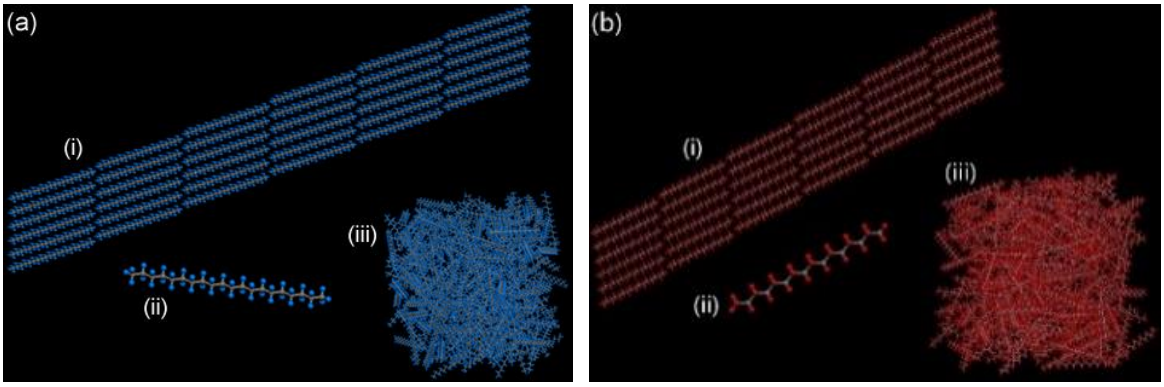

Figure S3. The crystal structures of (a) *n*-eicosane and (b) *n*-hexadecane, where (i) is the solid form, (ii) is the single molecule, and (iii) is the liquid form.

Table S1. The calculated free energies for solid and liquid *n*-hexadecane molecules with different calculation steps.

N-hexadecane	3000 steps (kcal mol^{-1})	5000 steps (kcal mol^{-1})
Solid	-4.20×10^3	-4.21×10^3
Liquid (50Å×50Å×50Å)	-1.56×10^3	-1.71×10^3
Liquid (55Å×55Å×55Å)	-1.35×10^3	-1.54×10^3
Liquid (60Å×60Å×60Å)	-8.91×10^2	-1.12×10^3

Table S2. The calculated free energies for solid and liquid *n*-eicosane molecules with different calculation steps.

N-eicosane	3000 steps (kcal mol^{-1})	5000 steps (kcal mol^{-1})
Solid	-5.24×10^3	-5.24×10^3
Liquid (50Å×50Å×50Å)	-1.54×10^3	-1.80×10^3
Liquid (55Å×55Å×55Å)	-1.13×10^3	-1.46×10^3
Liquid (60Å×60Å×60Å)	-1.10×10^3	-1.38×10^3

References

1. Fu, D. S.; Su, Y. L.; Gao, X.; Liu, Y. F.; Wang, D. J. Wang, D. J. Confined Crystallization of *n*-Hexadecane Located inside Microcapsules or outside Submicrometer Silica Nanospheres: A Comparison Study. J. Phys. Chem. B, 2013, 117, 6323–6329.

2. Kraack, H.; Sirota, E. B.; Deutsch, M. Measurements of Homogeneous Nucleation in Normal-Alkanes. J. Chem. Phys. 2000, 112, 6873.

3. Zerze, H.; Mittal, J.; McHugh, A. Hugh, A. J. Ab Initio Crystallization of Alkanes: Structure and Kinetics of Nuclei Formation. J. Macromolecules 2013, 46, 9151−9157.
